# Supplementary material for: Saccharomyces boulardii (CNCM I-745) improves intestinal damage in sepsis by remodeling intestinal flora structure
Source: Front Cell Infect Microbiol. 2025 Jul 31;15:1602792. doi: 10.3389/fcimb.2025.1602792 (PMC12350329; doi:10.3389/fcimb.2025.1602792)
Supplement: Supplementary file 1 [file DataSheet1.zip › summary/summary/8_Advanced_analyse/2_PICRUSt2_result/COG/Group.CW0_vs_AW0/PICRUSt2_COG_Group.CW0_vs_AW0_diff.pdf]

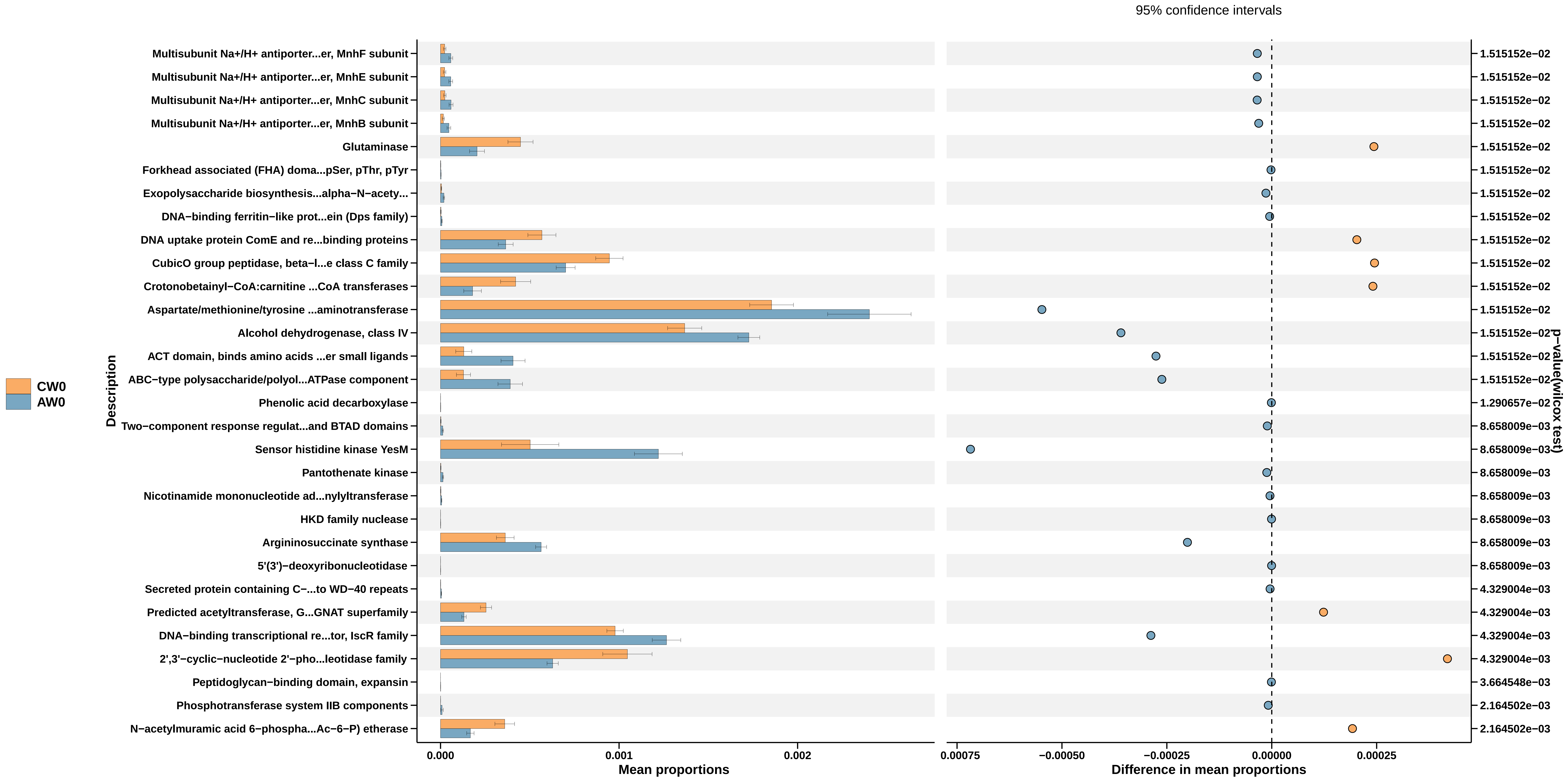

95% confidence intervals
CW0
AW0
Description
Multisubunit Na+/H+ antiporter...er, MnhF subunit
Multisubunit Na+/H+ antiporter...er, MnhE subunit
Multisubunit Na+/H+ antiporter...er, MnhC subunit
Multisubunit Na+/H+ antiporter...er, MnhB subunit
Glutaminase
Forkhead associated (FHA) doma...pSer, pThr, pTyr
Exopolysaccharide biosynthesis...alpha−N−acety...
DNA−binding ferritin−like prot...ein (Dps family)
DNA uptake protein ComE and re...binding proteins
CubicO group peptidase, beta−l...e class C family
Crotonobetainyl−CoA:carnitine ...CoA transferases
Aspartate/methionine/tyrosine ...aminotransferase
Alcohol dehydrogenase, class IV
ACT domain, binds amino acids ...er small ligands
ABC−type polysaccharide/polyol...ATPase component
Phenolic acid decarboxylase
Two−component response regulat...and BTAD domains
Sensor histidine kinase YesM
Pantothenate kinase
Nicotinamide mononucleotide ad...nylyltransferase
HKD family nuclease
Argininosuccinate synthase
5'(3')−deoxyribonucleotidase
Secreted protein containing C−...to WD−40 repeats
Predicted acetyltransferase, G...GNAT superfamily
DNA−binding transcriptional re...tor, IscR family
2',3'−cyclic−nucleotide 2'−pho...leotidase family
Peptidoglycan−binding domain, expansin
Phosphotransferase system IIB components
N−acetylmuramic acid 6−phospha...Ac−6−P) etherase
0.000
0.001
0.002
Mean proportions
0.00075
−0.00050
−0.00025
0.00000
0.00025
Difference in mean proportions
p−value(wilcox test)
1.515152e−02
1.515152e−02
1.515152e−02
1.515152e−02
1.515152e−02
1.515152e−02
1.515152e−02
1.515152e−02
1.515152e−02
1.515152e−02
1.515152e−02
1.515152e−02
1.515152e−02
1.515152e−02
1.515152e−02
1.290657e−02
8.658009e−03
8.658009e−03
8.658009e−03
8.658009e−03
8.658009e−03
8.658009e−03
8.658009e−03
4.329004e−03
4.329004e−03
4.329004e−03
4.329004e−03
3.664548e−03
2.164502e−03
2.164502e−03
